# A Novel High‐Pressure Tin Oxynitride Sn_2_N_2_O

**DOI:** 10.1002/chem.201904529

**Published:** 2020-01-22

**Authors:** Shrikant Bhat, Leonore Wiehl, Shariq Haseen, Peter Kroll, Konstantin Glazyrin, Philipp Gollé‐Leidreiter, Ute Kolb, Robert Farla, Jo‐Chi Tseng, Emanuel Ionescu, Tomoo Katsura, Ralf Riedel

**Affiliations:** ^1^ Photon Science DESY Notkestrsse 85 22607 Hamburg Germany; ^2^ FB Material- und Geowissenschaften Technische Universität Darmstadt 64287 Darmstadt Germany; ^3^ Department of Chemistry and Biochemistry The University of Texas at Arlington Arlington Texas 76019-0065 USA; ^4^ Institut für Physikalische Chemie Johannes Gutenberg-Universität Mainz 55128 Mainz Germany; ^5^ Bayerisches Geoinstitut (BGI) University of Bayreuth 95440 Bayreuth Germany

**Keywords:** density functional calculations, electron diffraction, high-pressure synthesis, synchrotron radiation, tin oxynitride

## Abstract

We report the first oxynitride of tin, Sn_2_N_2_O (SNO), exhibiting a Rh_2_S_3_‐type crystal structure with space group *Pbcn*. All Sn atoms are in six‐fold coordination, in contrast to Si in silicon oxynitride (Si_2_N_2_O) and Ge in the isostructural germanium oxynitride (Ge_2_N_2_O), which appear in four‐fold coordination. SNO was synthesized at 20 GPa and 1200–1500 °C in a large volume press. The recovered samples were characterized by synchrotron powder X‐ray diffraction and single‐crystal electron diffraction in the TEM using the automated diffraction tomography (ADT) technique. The isothermal bulk modulus was determined as *B*_o_=193(5) GPa by using in‐situ synchrotron X‐ray diffraction in a diamond anvil cell. The structure model is supported by DFT calculations. The enthalpy of formation, the bulk modulus, and the band structure have been calculated.

## Graphical abstract

**Tin hits six**: High‐pressure synthesis of the first tin oxynitride Sn_2_N_2_O (SNO) is reported. All “Sn” atoms are in six‐fold coordination, showing a Rh_2_S_3_‐type crystal structure with space group *Pbcn*. The results were also confirmed by DFT calculations (see figure).

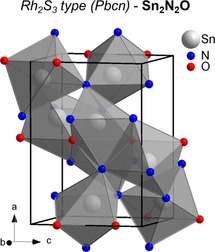

## Introduction

The Group 14 elements Si, Ge, and Sn form well‐known nitrides^1^ with exceptional thermomechanical and optoelectronic properties. Low compressibility and high hardness1a, 2 have been reported for spinel‐type Si and Ge nitrides, and their solid solutions are predicted to form a new family of wide direct band gap semiconductors.^3^ Hitherto, oxynitrides are known for Si and Ge, but not for Sn. Silicon oxynitride (Si_2_N_2_O), named “Sinoite”, was discovered in a meteorite by Andersen et al.1e in 1962. At the same time, a synthetic Si_2_N_2_O was prepared by nitriding a mixture of silicon and quartz powder at 1450 °C.1f It shows orthorhombic symmetry with space group *Cmc2*_1_.1f Sinoite proved to be one of the most stable, oxidation resistant refractory oxynitrides.^4^ Its formation and thermodynamic stability at ambient and high pressure have been investigated computationally previously.1c An isostructural germanium oxynitride Ge_2_N_2_O has been synthesized by ammonolysis of germanium oxides at temperatures above 850 °C as well.1d, 5

An oxynitride of tin is still unknown and has to the best of our knowledge not been reported as bulk material. There are a few reports on thin films of SnO_*x*_N_*y*_,^6^ described as nitrogen‐doped tin oxide with undetermined chemical composition and with a crystal structure related to SnO_2_, either as rutile‐type (mineral name cassiterite), or as pyrite‐type high‐pressure form.6a, 7 We did not find any computational studies related to tin oxynitrides.

Herein, we report the first tin oxynitride, exhibiting a Rh_2_S_3_‐type crystal structure, which is known as a high‐pressure modification of several oxides including Al_2_O_3_ and Fe_2_O_3_. All Sn atoms are in six‐fold coordination, in contrast to the sinoite‐type with only four‐fold metal coordination. We employed a high‐pressure high‐temperature (HP‐HT) method, using a large volume press (LVP) for the synthesis of a novel Sn_2_N_2_O compound (SNO). Materials are characterized by elemental analysis, angle‐dispersive XRD, TEM in combination with automated diffraction tomography (ADT), and energy‐dispersive X‐ray spectroscopy (EDX). We also determined the bulk modulus of SNO and used DFT calculations for structure modelling.

## Results and Discussion

### Precursor and HP‐HT synthesis

The XRD pattern of the Sn‐N‐O precursor, prepared by low temperature ammonolysis, shows spinel‐type Sn_3_N_4_ (as the only crystalline phase) and an amorphous background (Figure S1, Supporting Information). Elemental analysis by the combustion method confirmed a content of 5.3(3) wt.% oxygen and 11.2(2) wt.% nitrogen in the synthesized compound. From these values, elemental contents of 18 (1) at. % O, 43.6 (8) at. % N, and 38(1) at. % Sn in the as‐received compound can be calculated, which may be described as a mixture of tin nitride and tin oxide with a molar ratio of 1:1. This formally corresponds to a Sn_2_N_2_O stoichiometry. This precursor was subjected to HP‐HT conditions (20 GPa and 1200–1500 °C) in a Hall‐type six‐ram LVP (mavo press LPQ6 1500‐100; Max Voggenreiter GmbH, Germany) installed at the P61B beamline at DESY, Hamburg. After HP‐HT treatment, each recovered sample was dense and fine‐grained in appearance (Figure S3, Supporting Information). EDX spectroscopy on the recovered samples confirms the presence of oxygen in addition to nitrogen and tin (Figure S4, Supporting Information) without other contamination. Quantification of O and N was not possible as EDX is not reliable for low Z elements.^8^

### X‐ray diffraction

**Figure 1:**
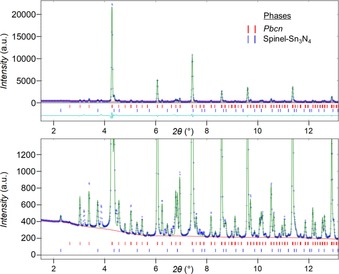
XRD pattern of SNO, in full (top) and magnified by a factor of ≈20 (bottom), with Rietveld refinement (green line) in space group *Pbcn* (*λ=*0.20736 Å). The reflection markers show the calculated reflection positions of the SNO phase (upper row, in red online) and the spinel Sn_3_N_4_ (lower row, in blue online).

The XRD pattern of the HP‐HT product measured with synchrotron radiation (Figure 1) shows a manifold of well resolved reflections—apart from a small fraction of spinel Sn_3_N_4_—which do not match to any known phase in the powder diffraction database (ICDD). They represent a novel phase, which (in the first approach) could be indexed by a primitive cubic lattice with *a=*7.831(2) Å. A preliminary structure model of SNO could be deduced from the integrated intensities of the powder reflections, using direct methods (shelxs2013^9^). This model in space group *Pa* $\bar{3}$ represents a double perovskite with two inequivalent Sn sites. One Sn shows a distorted octahedral coordination as usual in low‐symmetry double perovskites. The other Sn resides in the centre of eight corner‐linked octahedra, a position which in perovskites usually is occupied by atoms larger than those in the octahedral position. The cation to anion ratio of 2:3, implied by the structure model, is in accordance with the measured chemical composition Sn_2_N_2_O (for Sn^4+^, N^3−^, O^2−^). However, the model appears to represent only an averaged structure. No satisfying Rietveld refinement could be achieved within the framework of the cubic unit cell. On the other hand, a possible splitting of X‐ray reflections indicating a distortion of the cubic unit cell to lower symmetry seems to be too small to be detected in the XRD pattern.

### Electron diffraction tomography (ADT)

To elucidate the correct crystal symmetry of SNO, electron diffraction covering the 3D space was performed, namely automated diffraction tomography (ADT)^10^ on a synthesized sample (run# HH148 at 20 GPa, 1500 °C, Ta capsule). This sample contained only a very low fraction of spinel Sn_3_N_4_ and no third phase, as confirmed by XRD (CuKα). ADT allows for collecting electron‐diffraction intensities from single crystals as small as some tens of nm, and thus enables single‐crystal structure determination from such small crystals. Crystals were used without any special orientation to the electron beam. The acquired off‐zone diffraction patterns show reduced dynamical scattering effects and provide intensity data suitable for kinematical structure determination approaches like direct methods. For data acquisition a series of electron diffraction images (2D detector) are collected while tilting the crystal by 1° after every image. The wedges between the zones are integrated using electron beam precession. By processing these images afterwards, all patterns are reconstructed into a 3D diffraction space allowing visualization of the full diffraction information. More details are shown in the Supporting Information.

The unit‐cell was derived from ADT data with lattice parameters *a=*7.80, *b=*5.42, *c=*5.52 Å, which corresponds to an orthorhombic metric in the limit of expected errors of 1 % for cell axes and 1° for angles. Two of the cell edges are approximately equal in length, and by a factor of √2 shorter than the third one, in accordance with the pseudo‐cubic unit cell from the XRD powder pattern. Inspection of the systematic extinctions leads to a match with space group (S.G.) *Pbcn*. The crystal structure was solved from 224 unique diffraction intensities at a resolution limit of 0.8 Å, using direct methods (SIR2014).^11^ For the further evaluation of the single‐crystal ADT measurement, optimized lattice parameters from the synchrotron powder XRD data were used, as they are much more precise than those from electron diffraction.

### Lattice parameters from XRD

The lattice parameters of SNO were optimized by Rietveld refinement of the synchrotron powder data in S.G. *Pbcn*, with starting values from electron diffraction. In comparison with LaB_6_, only a small line broadening of the SNO reflections was observed, corresponding to a crystallite size of ≈250 nm. The refined lattice parameters are *a=*7.8257(8), *b=*5.5315(5), *c=*5.5438(4), and *V=*239.98(4) Å^3^ with ratios of *a*/*b=*1.4148=1.0004*x* √2 and *a*/*c=*1.4116=0.9982*x* √2. The deviation of the unit‐cell parameters from pseudo‐cubic symmetry is so small, that the orthorhombic line splitting is less than the reflection width (FWHM) in the synchrotron powder diagram, as shown by the red tic marks in Figure 1. The upper figure shows the full XRD pattern with the profile calculated by Rietveld refinement in green colour and the difference curve in cyan. Below, an enlarged view of the same pattern is shown, magnified by a factor of ≈20 to enable visualization of the weak reflections. The structure model in space‐group *Pbcn* was adopted from the crystal structure derived from ADT and not refined. The final *wR* is 7.5 % and the phase fraction of spinel Sn_3_N_4_ (blue tic marks) refined to 8.6 wt. %. There remain a few noninterpreted reflections belonging to a third, yet unknown phase (visible XRD data points without green calculated profile). These reflections cannot be explained by metallic tin or any of the reported Sn−O phases such as SnO_2_ at ambient conditions (rutile‐type *P*4_2_/*mnm*)^12^ or at high‐pressure (*Pnnm* and pyrite‐type *Pa* $\bar{3}$ ),^7^ or by Sn_2_O_3_ (P1),^13^ Sn_3_O_4_ (*P*2_1_/*c*),^14^ or SnO (litharge‐type *P*4*/nmm*).^15^

### Crystal structure from ADT

**Figure 2:**
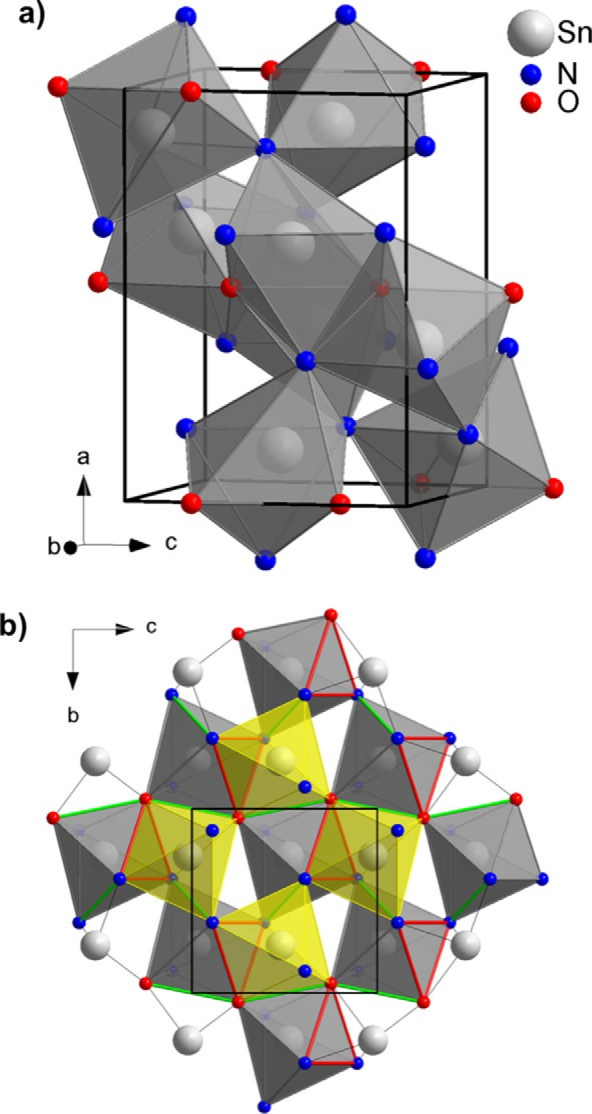
Crystal structure of SNO. a) Unit cell, showing the distorted octahedral coordination of Sn. b) View along the a‐axis, showing grey and yellow coloured octahedra sharing faces (indicated as red triangles). Additional edge sharing (indicated as green lines) connects octahedra into chains running along the *b*‐axis.

The crystal structure was kinematically refined from 5176 single‐crystal reflections measured as ADT data set up to a resolution of 0.5 Å. Averaging equivalent reflections in space group *Pbcn* resulted in 1046 unique reflections with *R*_int_=0.21. The final residual is *R*1=0.26. Dynamical refinement reduced the residual further to *R*1=0.09. Dynamically derived atomic positions are shown in Table 1, all atomic positions are provided in the Supporting Information. Tin and nitrogen atoms are in general position (8d), whereas oxygen is positioned on the 2‐fold axis (4c). The electron‐scattering factors of nitrogen and oxygen are too similar to distinguish these elements from the diffraction data. However, only the chosen assignment of sites represents an ordered structure, as the amounts of nitrogen and oxygen have a ratio of 2:1 and also the numbers of atomic positions of the sites 8d and 4c have a ratio of 2:1. There is no reason to assume nitrogen and oxygen to be disordered over both sites. Optimization of *Pbcn*‐Sn_2_N_2_O at ambient pressure using DFT confirms the soundness of the proposed structure of SNO. The calculated lattice parameters are *a=*7.83010, *b=*5.66335, *c=*5.62591 Å, (PBE values) and atomic coordinates are included in Table 1. The crystal structure of SNO is shown in Figure 2. All Sn atoms are symmetrically equivalent and have identical coordination, differing only by orientation or mirror symmetry. Within a single SnN_4_O_2_ octahedron the Sn−N and Sn−O bond lengths from experiment and theory are compared in Table 2. In the dense SNO structure the octahedra share edges and even faces, leading to short Sn−Sn distances of 3.269(5) and 3.091(7) Å, respectively.

**Table 1:** Atomic positions of SNO in space group *Pbcn* (No. 60) from experiment and DFT calculations.

Experiment (dynamical refinement)				
Atom	*x*	*y*	*z*	*U*_iso_ [Å^2^]
Sn1	0.11535(9)	0.24354(15)	0.52443(16)	0.0040(3)
N1	0.3507(5)	0.3841(7)	0.3952(9)	0.0012(8)
O1	0	0.4513(10)	0.25	0.0037(11)

DFT calculations				
Atom	*x*	*y*	*z*	
Sn1	0.1157	0.2376	0.5251	
N1	0.3507	0.3790	0.3954	
O1	0	0.4530	0.25

**Table 2:** Bond distances of Sn_2_N_2_O from experiment in comparison to DFT calculations.

Bond distance	DFT	Experiment	SnN_4_O_2_ octahedron
	*d* [Å]	*d* [Å]	with labelling of atoms
Sn−O(a)	2.169	2.110(3)	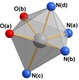
Sn−O(b)	2.343	2.286(4)	
Sn−N(a)	2.202	2.190(5)	
Sn−N(b)	2.174	2.130(4)	
Sn−N(c)	2.136	2.124(4)	
Sn−N(d)	2.223	2.233(4)	

### Equation of state

**Figure 3:**
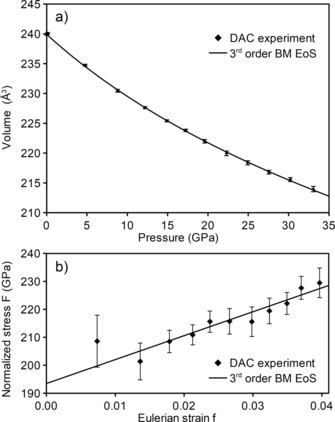
a) Pressure dependence of the unit‐cell volume of SNO and the 3rd order Birch–Murnaghan EoS fitted to the data. b) The same EoS, rescaled to show the normalized stress *F* in dependence on Eulerian strain *f*. In the *fF* plot a 3rd order BM EoS displays as a straight line with *y*‐axis intercept *B*_0_ and slope proportional to (*B*′*−*4).

The bulk modulus of SNO *B*_o_=193(5) GPa with pressure derivative *B*′=6.9(7) was determined from the pressure dependence of the unit‐cell volume by fitting a 3rd order Birch–Murnaghan equation of state (EoS). The pressure dependence of the unit‐cell volume is shown in Figure 3 a, while the slope (3/2 *B*_o_ (*B′−*4) ≠ 0) of the linear fF‐plot in Figure 3 b clearly indicates the Birch–Murnaghan equation of state to be of 3rd order rather than 2nd order (*B′*=4). The high value of the pressure derivative *B′* may be explained by deviatory stress, which can evolve in dense sintered powders with submicron grain size at high pressure even under a neon pressure medium.^16^ Deviatory stress shows up in the fF‐plot as a deviation of data points from a straight line in the high‐pressure range. In our sample, however, such deviations are smaller than the experimental errors (cf. Figure 3 b) and do not justify judging the highest‐pressure data points as significantly affected by deviatoric stress.

### DFT calculations

For Sn_2_N_2_O, we computed a bulk modulus *B_o_* of 177 and 208 GPa by using the PBE and SCAN functional, respectively. The experimentally determined bulk modulus fits within this range. This is about 10 % higher than *B*_o_ of spinel‐type Sn_3_N_4_, which we computed to 164 GPa [SCAN: 191 GPa] (exp. 148±1.2 GPa).^17^ The shear modulus *G* is 95 GPa using the PBE functional [SCAN: 112 GPa], about 10 % lower than *G* of Sn_3_N_4_. Combining *B*_o_ and *G* yields a single‐crystal hardness of Sn_2_N_2_O of 11–12 GPa.^18^ Since *G* is lower and *B*_o_ is higher in comparison to Sn_3_N_4_, the hardness value of Sn_2_N_2_O can be expected to be 30–50 % lower than the hardness of Sn_3_N_4_ (14–17 GPa). Band structure calculations for Sn_2_N_2_O by PBE and SCAN yield gaps of 0.3 and 0.6 eV, respectively. These values likely underestimate the true band gap. Using hybrid functionals we find a gap of 1.3 and 1.9 eV for HSE06^19^ and PBE0^20^ calculations, respectively. Thus, we expect that the gap of Sn_2_N_2_O single crystals will be close to that of Sn_3_N_4_, for which a value of 1.6 eV has been calculated^21^ and 1.6±0.2 eV3b determined experimentally.

**Figure 4:**
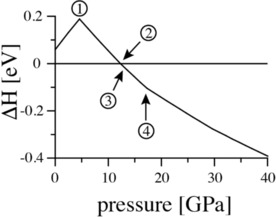
Reaction enthalpy (PBE results) of the reaction SnO_2_+Sn_3_N_4_ → 2 Sn_2_N_2_O as a function of pressure. The labels indicate (1) transformation of spinel‐type Sn_2_N_2_O to *Pbcn*‐ Sn_2_N_2_O (at 4.6 GPa), (2) Δ*H*=0 (12.0 GPa), (3) transformation of rutile‐type SnO_2_ to α‐PbO_2_‐type SnO_2_ (12.5 GPa), and (4) transformation of α‐PbO_2_‐type SnO_2_ to pyrite‐type SnO_2_ (17.2 GPa).

To rationalize the formation of Sn_2_N_2_O in the high‐pressure experiment we computed its enthalpy of formation from the binaries SnO_2_ and Sn_3_N_4_ as a function of pressure. SnO_2_ itself displays a rich high‐pressure chemistry and has been subject of multiple experimental and computational studies.^7^, ^22^ At low pressure the rutile‐type of SnO_2_ is most favourable. We compute the pressure at which it transforms into the pyrite‐type of SnO_2_ to 9.6 GPa. Sn_3_N_4_ adopts the spinel structure at ambient pressure and transforms into post‐spinel phases only above 40 GPa. Huang et al.^23^ predicted CaTi_2_O_4_‐ and CaFe_2_O_4_‐type orthorhombic structures at 40 and 60 GPa, respectively. On the other hand, Kearney et al.^24^ predicted structures with space groups *P*2_1_/*c* and *R* $\bar{3}$ *c* at pressures above 40 and 87 GPa, respectively. At ambient pressure, the enthalpy of formation Δ*H*_f_ of *Pbcn*‐Sn_2_N_2_O from the binaries is +0.56 eV/Sn_2_N_2_O; taking the more favourable (at zero pressure) defect‐spinel‐type of Sn_2_N_2_O the enthalpy difference is still +0.30 eV/Sn_2_N_2_O. This indicates that Sn_2_N_2_O is *meta*‐stable with respect to decomposition into the binaries. Figure 4 shows that Sn_2_N_2_O will form at 12.0 GPa [SCAN: 14.3 GPa] and remains stable against decomposition at higher pressures.

Searching for alternative structures of Sn_2_N_2_O potentially appearing at low or high pressures, we found two relevant candidates. A “defect” spinel‐type structure with disorder among the anions and vacancies on cation sites, constitutes the low‐pressure modification of Sn_2_N_2_O. While we computed the lowest energy structure to adopt the ordered spinel In_2_S_3_‐type structure, we found other “defective” variants of spinel (e.g. with S.G. *P*3_2_ *m*1) within a few meV/atom. All “defect” spinel‐types exhibit tetrahedrally and octahedrally coordinated cations. At ambient pressure, the “defect” spinel‐type of Sn_2_N_2_O is about 0.25 eV/Sn_2_N_2_O more favourable than the *Pbcn*‐type. The transition to *Pbcn*‐Sn_2_N_2_O occurs at 5 GPa. The newly synthesized tin oxynitride remains the most favourable structure up to 95 GPa, when a new Sn_2_N_2_O‐III with seven‐ and eight‐fold coordinated cations (CN=7.5 at 100 GPa) and orthorhombic (*Pmn*2_1_) symmetry becomes favoured by enthalpy. Sn_2_N_2_O‐III distorts upon decompression into a monoclinic (*P*2_1_) structure with reduced coordination.

Re‐investigating the sequence of high‐pressure structures of Si_2_N_2_O1c we find that a *Pbcn*‐Si_2_N_2_O will succeed the Al_2_O_3_‐type only above 90 GPa. The Rh_2_S_3_‐type structure (sometimes addressed as Rh_2_O_3_‐II type) with space group *Pbcn* is known as a high‐pressure phase of α‐Al_2_O_3_ (corundum, *R* $\bar{3}$ *c*) above 113 GPa,^25^ or α‐Fe_2_O_3_ (hematite, *R* $\bar{3}$ *c*) above 41 GPa,^26^ or Bixbyite‐type In_2_O_3_ in the range of 10–25 GPa,^27^ in which the latter compound shows also a metastable corundum‐type phase at ≈6 GPa.

## Conclusions

In conclusion, the discovered tin oxynitride Sn_2_N_2_O with space group *Pbcn* (Rh_2_S_3_‐type structure) is another example demonstrating the potential of high‐pressure synthesis to access novel materials. Tin is in six‐fold coordination in the Rh_2_S_3_‐type structure in contrast to the sinoite‐type oxynitrides of Si and Ge with four‐fold metal coordination. Tin oxynitride was synthesized at high pressure and high temperature using a large volume press at beamline P61B, DESY, Hamburg. The compound forms from tin oxide and tin nitride only at high pressure and can be quenched to ambient pressure retaining its high‐pressure structure. The crystal structure of the novel compound was determined using synchrotron powder XRD in combination with single‐crystal electron diffraction tomography from a 200 nm sized single crystal. DFT calculations predicted this structure to be stable above 12 GPa and to remain the most favourable one up to 95 GPa. The calculations also confirmed the ordered distribution of nitrogen and oxygen. The synthesized tin oxynitride may be considered for applications, for example, in the field of low band‐gap semiconductors. From the viewpoint of fundamental solid‐state chemistry, Sn_2_N_2_O is a new member of inorganic compounds with the well‐known Rh_2_S_3_‐type structure. It is also the first evidence for the existence of a crystalline ternary oxynitride of tin and complements and extends the well‐known Group 14 element oxynitrides of silicon and germanium.

## Experimental Section

### Synthesis

Oxygen‐containing tin nitride precursors were synthesized from a Sn[NMe_2_]_4_ complex using a two‐stage ammonolysis procedure as described by Hector et al.^28^ In the first stage, 7.93 mmol of Sn[N(CH_3_)_2_]_4_ was dissolved in 50 mL THF and cooled to −78 °C. Then, condensed ammonia was added by dropping method to the solution under constant stirring. A yellow powder was recovered after drying from the solvent and subsequently subjected to a second‐stage standard ammonolysis at 150 °C for 6 h and then to 400 °C for 2 h (heating rate 2 °C min^−1^; details are given in the Supporting Information). The product was characterized by XRD and elemental analysis using the combustion method (LECO tester). This precursor was subjected to HP‐HT conditions (20 GPa and 1200–1500 °C) in a Hall‐type six‐ram LVP (mavo press LPQ6 1500‐100; Max Voggenreiter GmbH, Germany) installed at the P61B beamline at DESY, Hamburg.

### XRD

X‐ray diffraction of a crushed, synthesized sample (run# HH127, 20 GPa, 1200 °C, Pt capsule) was performed using synchrotron radiation at the high‐resolution powder beamline P02.1 of PETRA‐III, DESY, Hamburg. The XRD pattern was quantitatively analysed by Rietveld refinement with the program GSAS‐II^29^ using instrumental profile parameters from a LaB_6_ standard measured on the same instrument. A second sample (run# HH148, 20 GPa, 1500 °C, Ta capsule) was characterized with CuKα radiation from a laboratory source. The pressure dependence of the unit‐cell volume of SNO was measured in a diamond anvil cell (DAC) using synchrotron radiation at the extreme‐condition beamline P02.2 of PETRA III, DESY, upon increasing the pressure from 0.14 to 33 GPa. The pressure in the DAC was measured with the ruby fluorescence method. The bulk modulus and its pressure derivative was determined by an equation of state fit [EosFit v5.2].^30^ The volume at ambient pressure, *V*_0_=239.80(1) Å^3^, was determined from the Rietveld refinement of an X‐ray pattern measured with CuKα radiation using a sample from the same HP‐HT run (#HH141, 20 GPa, 1300 °C, Ta capsule) as for the DAC experiment.

### Electron diffraction (ADT)

Automated diffraction tomography (ADT) experiments^10^ were carried out at the electron microscopy centre (EMC‐M) of the Johannes Gutenberg‐University Mainz. A TEM Tecnai F30 ST equipped with a field emission gun at 300 kV was used. Sample imaging for crystal tracking during stage tilt was performed in μ‐STEM mode with a HAADF detector. Diffraction measurements were performed using nanoelectron‐diffraction (NED) mode with a 10 μm C2 aperture leading to a semi‐parallel illumination with a beam size of 200 nm. Electron diffraction patterns were acquired with a Gatan CCD US4000 using 2k *x* 2k images taken with 1 s exposure time for each pattern. To provide a high tilt range a Fischione tomography sample holder was used (tilt range of −45–60° due to limitations on the grid). ADT data were collected with electron beam precession (precession electron diffraction, PED) to improve reflection intensity integration quality using a Digistar unit developed by NanoMEGAS SPRL (precession angle 1°).^31^ A fast acquisition module recently developed was used for diffraction data acquisition applying sequential tilts of 1°. The Fast‐ADT acquisition is reported in more detail in the upcoming article of Plana‐Ruiz et al.^32^ Data processing, 3D reconstruction, cell parameter determination and space group analysis as well as diffraction intensity extraction were performed with ADT3D (eADT) software.^33^ Ab initio structure solution assuming the kinematic approximation (Int.∝|F(hkl)|^2^) was performed by direct methods implemented in the program SIR2014 including as well difference Fourier mapping and least‐squares refinement.^11^ Scattering factors for electrons were taken from Doyle and Turner.^34^ Kinematical structure refinement was performed with shelxl2013^9^ and dynamical structural refinement with JANA2006^35^ after data extraction with PETS.^36^

### DFT calculations

Calculations of total energy and volume were done within density functional theory as implemented in the Vienna ab initio simulation package (VASP).^37^ The Generalized Gradient Approximation (GGA‐PBE)^38^ was used together with the projector‐augmented‐wave (PAW) method.^39^ Consistency checks were done with the Strongly Conserved and Appropriately Normed (SCAN) functional.^40^ All results were obtained using a plane wave cut‐off energy of 500 eV with forces converged to better than 1 meV Å^−1^. The Brillouin zone of each structure was sampled by k‐point meshes with grid sizes smaller than 0.03 Å^−1^. With the parameters above, enthalpy differences between structures are converged to better than 1 meV. Enthalpy and pressure of products and reactants are extracted from energy‐volume data of corresponding structures. Computed elastic constants yield Young's modulus *E* and shear modulus *G* via the Voigt–Reuss–Hill approximation.^41^ The approach of Chen et al.^18^ was used to estimate single‐crystal hardness. Searching for additional and alternative potential candidate structures of Sn_2_N_2_O we applied the USPEX code.^42^ The code readily produced *Pbcn*‐Sn_2_N_2_O and *Pmn*2_1_‐Sn_2_N_2_O among a manifold of structures. Low‐pressure modifications of defective spinel‐types were taken from previous works1c, 43 and outperformed models provided by the search algorithm.

## Conflict of interest

The authors declare no conflict of interest.

## Supporting information

As a service to our authors and readers, this journal provides supporting information supplied by the authors. Such materials are peer reviewed and may be re‐organized for online delivery, but are not copy‐edited or typeset. Technical support issues arising from supporting information (other than missing files) should be addressed to the authors.

Supplementary
