# Supplementary material for: A Novel High‐Pressure Tin Oxynitride Sn2N2O
Source: Chemistry. 2020 Jan 22;26(10):2187–94. doi: 10.1002/chem.201904529 (PMC7065226; doi:10.1002/chem.201904529)
Supplement: Supplementary file 1 — Supplementary [file CHEM-26-2187-s001.pdf]

CHEMISTRY
A European Journal

Supporting Information

A Novel High-Pressure Tin Oxynitride Sn_2N_2O

Shrikant Bhat,*[a, e] Leonore Wiehl,[b] Shariq Haseen,[c] Peter Kroll,[c] Konstantin Glazyrin,[a] Philipp Gollé-Leidreiter,[b] Ute Kolb,[b, d] Robert Farla,[a] Jo-Chi Tseng,[a] Emanuel Ionescu,[b] Tomoo Katsura,[e] and Ralf Riedel[b]

chem_201904529_sm_miscellaneous_information.pdf

Supporting Information

Table of contents:

1. Preparation of starting materials
2. HP-HT syntheses using the large volume press
3. Single crystal structure determination from electron diffraction in the TEM
4. Bulk modulus
5. Technical details of DFT calculations

1. Preparation of starting materials:

Oxygen bearing Sn_3N_4 precursors were produced by ammonolysis of tetrakis(dimethylamino)tin, $Sn[N(CH_3)_2]_4$. We used a two-stage ammonolysis procedure as described by Hector et al.[1] $Sn[N(CH_3)_2]_4$ (7.93mmol) was dissolved in 50 ml THF and cooled to -78 °C. Then, condensed ammonia was added via dropping method to the solution under constant stirring. We have also noticed the formation of a xerogel during this procedure as mentioned earlier[1]. Once the gel formation was complete, the ammonia flow was stopped. The gel was allowed to warm up slowly to ambient temperature with stirring, so that the excess ammonia evaporated leaving a slurry of pale-yellow powder. The yellow powder was recovered after drying from the solvent and subsequently subjected to a second stage standard ammonolysis at 150 °C for 6 h and then to 400 °C for 2 h (ramp 2 °C min^{-1}). The product was washed in air with 3 M HCl (aq) followed by 3 portions of ethanol and dried in air. XRD of the raw product shows spinel-type Sn_3N_4 (as the only crystalline phase) and an amorphous background (Figure S1).

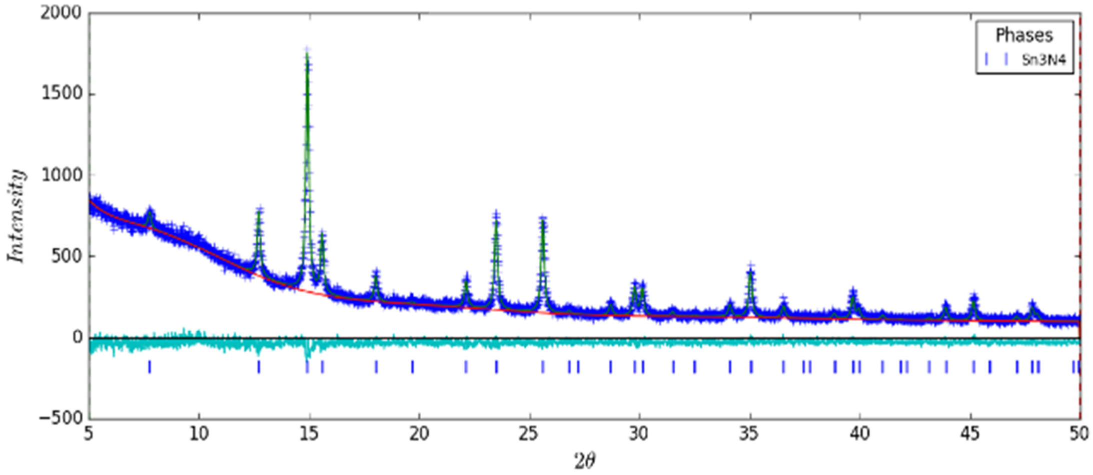

Fig. S1: XRD pattern (Mo Kα) of the as-synthesized precursor with Rietveld refinement of the spinel Sn_3N_4 phase

Nitrogen and oxygen content in the starting materials were analyzed using combustion analysis by a LECO analyzer. It is one of the reliable and reproducible methods used especially for inorganic compounds, for the determination of C, N, O contents with high accuracy[2]. The results showed 18.06 ±1.02 at% O and 43.59 ± 0.77 at% of N content in the synthesized compound.

2. HP-HT syntheses using the large volume press

HP-HT experiments were performed in a Hall-type six-ram press (mavo press LPQ6 1500-100; Max Voggenreiter GmbH, Germany) installed at the P61B beamline at DESY, Hamburg. Second stage anvils (tungsten carbide cubes, 32 mm Fujilloy TF08) with a truncated edge length of 4 mm were used. A Cr_2O_3-doped MgO octahedron with an edge length of 10 mm was used as the pressure transmitting medium. The starting material was cold pressed into pellet form (h = 1.4 mm, ø =1.8 mm) using a metal foil (Ta / Pt) before placing inside the MgO tube. The schematic diagram of the assembly is given in Figure S2. The octahedral assembly was compressed to a pressure of approximately 20 GPa and, at pressure, heated to a chosen temperature in the range of 1200 -1500 °C and held for 30 min. Pressure was calibrated at room temperature using the semiconductor to metal transition of GaP at 22.2 GPa [3]. Temperature was calibrated in a separate run using a W5%Re/W26%Re thermocouple (C-type). After pressure release, each recovered sample was dense and fine-grained in appearance. Figure S3 shows SEM micrographs of a recovered (run# HH127) sample pellet (left) and tin oxynitride crystals of varied dimensions (right).

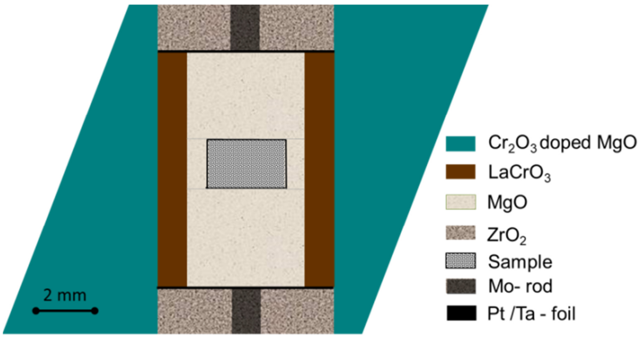

Fig. S2. Schematic diagram of the octahedron assembly

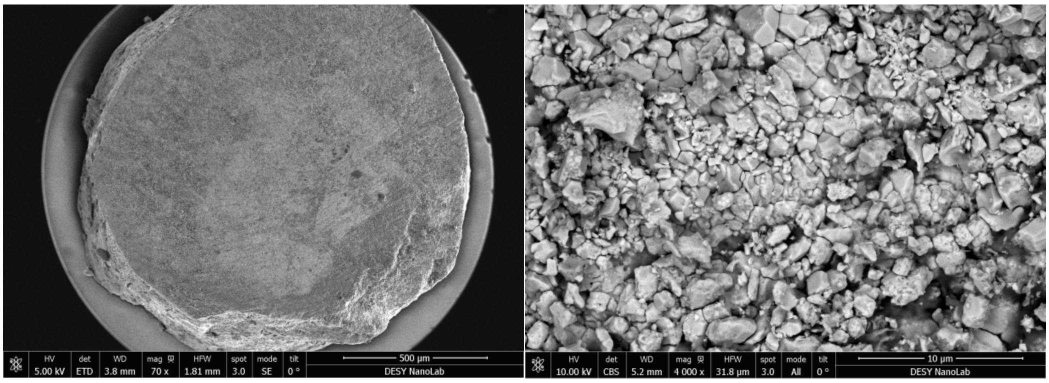

Fig. S3: SEM micrographs (run# HH127) showing an overview of the sample pellet after HP-HT (left) and tin oxynitride crystals of varied dimensions (right).

Three different tin oxynitride (SNO) samples were used for characterization, namely from run HH127 (20 GPa, 1200 °C, Pt capsule), from run #H141 (20 GPa, 1300°C, Ta capsule), and from run # HH148 (20 GPa, 1500°C, Ta capsule). X-ray diffraction (XRD) of crushed samples was obtained using synchrotron radiation at the high-resolution powder diffraction beamline P02.1 of PETRA-III, DESY, Hamburg (SNO-HH127) and using a benchtop diffractometer with Cu Kα radiation, in the LVP laboratory P61B, DESY, Hamburg (SNO-HH141, SNO-HH148). In the X-ray pattern of SNO-HH127 some of remaining starting material (spinel Sn_3N_4) could be detected, but only ~1 wt% in SNO-HH141 or HH148. Sample SNO-HH127 was used for Rietveld refinement, SNO-HH148 for electron diffraction (ADT), and SNO-HH141 for determination of the bulk modulus.

3. Single crystal structure determination from electron diffraction in the TEM

For TEM measurements, the powdered samples were dispersed in ethanol using an ultrasonic bath and sprayed on a carbon-coated copper grid using a modified ultrasound sonifier[4]. Automated diffraction tomography (ADT) experiments[5] were carried out at EMC-M at Johannes Gutenberg-Universität Mainz.

At EMC-M ADT measurements were performed without and with electron beam precession (PED), using a transmission electron microscope (TEM) Tecnai F30 ST equipped with a field emission gun at 300 kV set up for mild illumination (gun lens 8 and spot size 6). In order to provide a high tilt range a tomography sample holder (Fischione, max. tilt +- 70°) was selected. Sample imaging for crystal tracking during stage tilt was performed in microprobe scanning TEM mode (µ-STEM) with high angular annular dark field (HAADF, Fischione; Emispec ES Vision software) detector. Measurements were performed using nano electron diffraction (NED) mode with a 10 µm C2 aperture leading to a semi-parallel illumination with a beam size of 100 nm. Electron diffraction patterns were acquired with a Gatan CCD US4000 4k x 4k, 16 bit (binning 2 leads to 2k x 2k data) and 1 sec exposure time for each pattern.

An elemental analysis was performed at EMC-DA in Darmstadt with a TEM JEOL 2100F by energy dispersive X-ray (EDX) spectroscopy at 200 kV in STEM mode with a HAADF and an Oxford EDX detector. An EDX measurement is exemplarily shown in Figure S4.

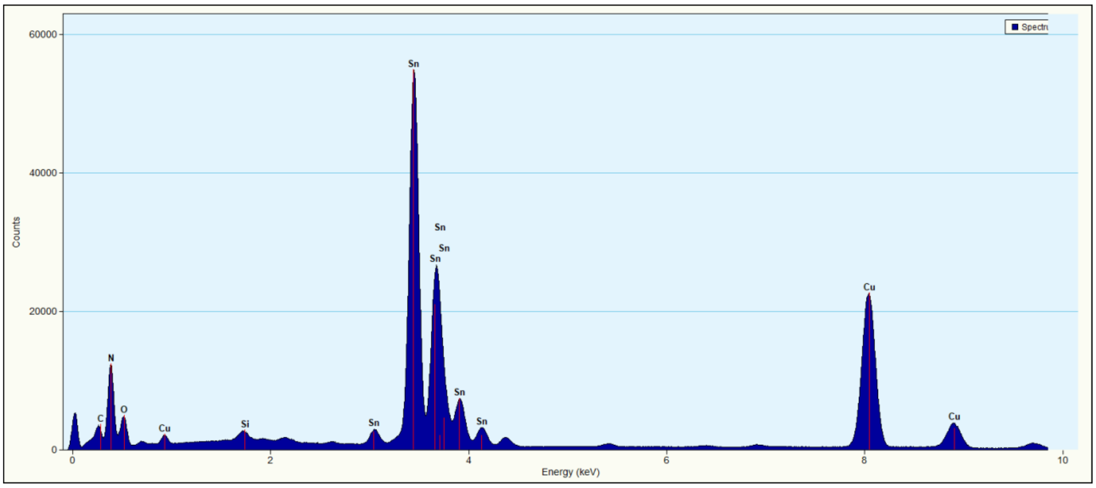

Figure S4. EDX spectrum as a point measurement in STEM. The sample was prepared on a carbon coated Cu-grid.

One of the crystals used for ADT measurement is shown exemplarily in Figure S5 as indicated by a red arrow. The crystal size is approximately 200 nm.

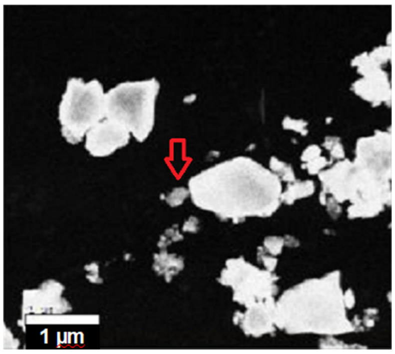

Fig.S5: The red arrow indiates the SNO single crystal used in the ADT measurement.

Data extraction was performed using a box size of 30x30 pixel and a spike length of 6. All data sets delivered comparable cell parameters within the expected error range of 1% for cell length and 1° degree for cell axes and showed the expected extinctions for space group *Pbcn* (see Figure S6).

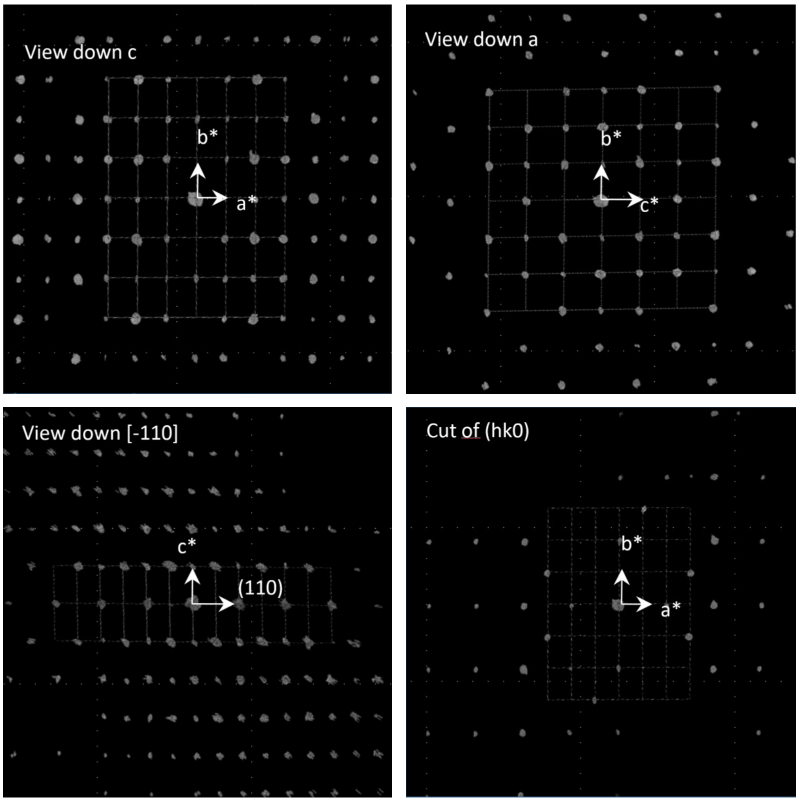

Fig.S6: Three-dimensional reconstruction of the reciprocal space based on ADT data. The diffraction volume viewed along the ***c***-axis (top left), the ***a***-axis (top right), the [-110] direction (bottom left) and the (hk0) reciprocal layer (bottom right), including the n-glide plane perpendicular to the **c**-axis show systematic extinctions compatible with space group *Pbcn*.

For structure solution cell parameters derived from powder XRD data (a = 7.8257(8) Å, b = 5.5315(5) Å, c = 5.5438(4) Å) were used. The diffraction data completeness of 90-100% allowed to solve the crystal structure with (SIR2014) by direct methods (@resolution 0.8 Å) based on the strongest three scattering density maxima in the solution. Crystal structure refinement was performed using SHELX with a limit of 0.5 Å resolution covering the full width of the CCD detectors. The refinement results of Cry_2 measured with PED and for comparison Cry_6 measured without PED are provided in Table S1 together with the values calculated by DFT. Additionally, results from dynamical refinerment of ADT data from Cry_2 are shown. In this case the program PETS was used for data extraction and JANA for refinement. Atomic positions are provided in Table S2. All atomic coordinates are compared in Table S3 including a figure showing the atom labeling.

Table S1: Crystallographic information on ADT measurements and single crystal structure refinement of SNO in space group *Pbcn*; kinematical with eADT/SHELX and dynamical with PETS/JANA in comparison with the DFT results.

	**DFT**	**Cry_6 (ADT)**	**Cry_2 (ADT/PED)**	**Cry_2 (ADT/PED)**
Titl range (deg)		±50°	-45° – +60°	-45° – +60°
**Refinement**		**Kinematic (SHELX)**	**Kinematic (SHELX)**	**Dynamic (JANA)**
a (Å)	7.83010	7.8257(8)	7.8257(8)	7.8257(8)
b (Å)	5.66335	5.5315(5)	5.5315(5)	5.5315(5)
c (Å)	5.62591	5.5438(4)	5.5438(4)	5.5438(4)
α (deg)	90	90	90	90
β (deg)	90	90	90	90
γ (deg)	90	90	90	90
V ($Å^3$)	249.48	239.98(4)	239.98(4)	239.98(4)
density (g/cm^3)		7.788	7.788	
wavelength (Å)		0.0197	0.0197	0.0197
Resolution (Å)		0.5	0.5	0.56
Θ_max (deg)		1.129	1.129	
measured reflections		4529	5176	6810
unique reflections		1004	1046	1706
unique reflections (I>2sig(I))		899	683	1272
Completeness (%)		100%	93%	94%
Overall Biso ($Å^2$)		0.49	0.64	
R(sigma)		0.142	0.117	
R(int)		0.264	0.208	
refined parameters		11	11	115
R1 (I>2sig(I))		0.318	0.256	0.088
Crystal thickness (Å)				212.5
Goodness of Fit		2.72	3.13	4.69
g_{max}				1.8
S_g^{max}(matrix),				0.01
R_{Sg},				0.35
N_{or}				300

Table S2. Atomic positions of Sn_2N_2O after refinement in SHELX (kinematic) or JANA (dynamic).

ADT (Cry_6) kinematic				
Atom	x	y	z	U(iso)
Sn	0.1152(4)	0.2418(3)	0.5238(5)	0.0076(7)
N	0.3539(8)	0.3804(16)	0.3925(16)	0.0028(11)
O	0	0.4598(19)	0.25	0.0030(12)
ADT/PED (Cry_2) kinematic				
Atom	x	y	z	U(iso)
Sn	0.1154(3)	0.2425(3)	0.5238(4)	0.0135(5)
N	0.3560(11)	0.3832(14)	0.4045(17)	0.0115(12)
O	0	0.444(3)	0.25	0.017(2)
ADT/PED (Cry_2) dynamic				
Atom	x	y	z	U(iso)
Sn	0.11535(9)	0.24354(15)	0.52443(16)	0.0040(3)
N	0.3507(5)	0.3841(7)	0.3952(9)	0.0012(8)
O	0	0.4513(10)	0.25	0.0037(11)

Table S3: Bond distances d (Å) with estimated standard deviations of Sn_2N_2O from different measurement in comparison to DFT calculations and their differences Δd (Å) from DFT and experiment. The labeling scheme is indicated in the octahedron figure.

	DFT	ADT (Cry_6)		ADT/PED (Cry2) kinematic		ADT/PED (Cry2) dynamic		SnN_4O_2 octahedron with labeling of atoms
Atoms	d (Å)	d (Å)	Δd (Å)	d (Å)	Δd (Å)	d (Å)	Δd (Å)	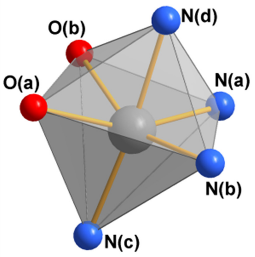
Sn - O(a)	2.169	2.138(6)	0.031	2.089(8)	0.080	2.110(3)	0.059	
Sn - O(b)	2.343	2.261(8)	0.082	2.323(11)	0.020	2.286(4)	0.057	
Sn - N(a)	2.202	2.167(10)	0.035	2.233(10)	-0.031	2.190(5)	0.012	
Sn - N(b)	2.174	2.141(9)	0.033	2.107(8)	0.067	2.130(4)	0.044	
Sn - N(c)	2.136	2.147(8)	-0.011	2.141(9)	-0.005	2.124(4)	0.012	
Sn - N(d)	2.223	2.203(7)	0.020	2.183(9)	0.040	2.233(4)	-0.010	

4. **Bulk modulus**

The pressure dependence of the unit cell volume is shown in Figure 3a, while the slope (≠ 0) of the linear fF-plot in Figure 3b clearly indicates the Birch-Murnaghan equation of state to be of 3rd order rather than 2nd order. B_0 was determined as 193(5) GPa with pressure derivative *B'* = 6.9(7).

The high value of the pressure derivative *B'* = 6.9(7) requires an additional clarification. We measured a piece of dense sintered powder with a grain of a submicron scale. We tried to avoid any additional treatment of the sample in order to prevent the sample from any additional transformation. It is known, that under the conditions of the strongly suppressed diffusion in a high-pressure environment of the diamond anvil cell experiment (uniaxial compression), even under a Neon pressure medium, one can expect evolution of the deviatoric stress for densely sintered samples. This stress and stress-strain field in the sample are enhanced by the developing micro-strains on the inter-grain boundaries upon the uniaxial compression. Additional information and a more detailed discussion can be found in Ref[6].

The evolving deviatoric stress always leads to a higher slope on the F-f plot (Fig. 3b). This is the effect of so called 'pressure overestimation'. As can be seen from the same figure, the slope, and thus the *B'* determination strongly depends on the range of the Eulerian strain or equally – the pressure range taken into the consideration. Since we have only limited understanding on the phase stability under various P-T conditions, thus, the additional treatment of the sample was out of question, and since it goes beyond the scope of this paper, we only note that the exact value for *B'* requires further investigation and has, most probably, lower value.

5. **Technical details of DFT calculations**

For relevant structures we compute energy E as a function of volume V, reducing V stepwise to simulate higher densities and pressures. The resulting E–V data yields pressure p by numerical differentiation, $p = -\partial E/\partial V$, and enthalpy $H = E + pV$. Reaction enthalpies ΔH as a function of pressure at zero temperature are computed based on enthalpies of products and reactants. Locating ΔH=0 then yields an estimate of transition pressures, neglecting further impact of temperature and of entropy differences to reaction Gibbs energies $\Delta G = \Delta H - T\cdot\Delta S$. These estimates are excellent guidelines predicting solid state reactions at constant composition, not at least because entropy differences that contribute to reaction Gibbs energies, ΔG for solid-state reactions are usually small in comparison to changes of ΔH within a few GPa of pressure.

Density of states (DOS) calculations use a 17x17x17 mesh and Blöchl's tetrahedron method [7] for calculations with the PBE and SCAN functionals. Using structures optimized in PBE with a 4x4x4 mesh, band structure calculations with the HSE06 and PBE0 hybrid functionals are done through the Γ-X-S-Y-Γ-Z-U-R-T-Z | Y-T | U-X | S-R path in the Brillouin zone to calculate band gaps. Charge density mixing is allowed for *s*-, *p*-, and *d*-electrons.

References

(1) Li, X.; Hector, A. L.; Owen, J. R.; Shah, S. I. U., Evaluation of nanocrystalline Sn3N4 derived from ammonolysis of Sn(NEt2)4 as a negative electrode material for Li-ion and Na-ion batteries. *J. Mater. Chem. A* **2016,** *4* (14), 5081-5087.
(2) Tessier, F., Determining the Nitrogen Content in (Oxy)Nitride Materials. *Materials* **2018,** *11* (8), 1331.
(3) Ono, S.; Kikegawa, T., Determination of the phase boundary of GaP using in situ high pressure and high-temperature X-ray diffraction. *High. Press. Res.* **2017,** *37* (1), 28-35.
(4) Mugnaioli, E.; Gorelik, T.; Kolb, U., "Ab initio" structure solution from electron diffraction data obtained by a combination of automated diffraction tomography and precession technique. *Ultramicroscopy* **2009,** *109* (6), 758-765.
(5) Kolb, U.; Gorelik, T.; Mugnaioli, E., Automated diffraction tomography combined with electron precession: a new tool for ab initio nanostructure analysis. *Mater. Res. Soc. Symp. Proc.* **2011**, 1184-GG01-05.
(6) Glazyrin, K.; Miyajima, N.; Smith, J. S.; Lee, K. K. M., Compression of a multiphase mantle assemblage: Effects of undesirable stress and stress annealing on the iron spin state crossover in ferropericlase. *J. Geophys. Res. Solid Earth* **2016,** *121* (5), 3377-3392.
(7) Blöchl, P. E.; Jepsen, O.; Andersen, O. K., Improved tetrahedron method for Brillouin-zone integrations. *Phys. Rev. B* **1994,** *49* (23), 16223-16233.
